# Autonomic regulation in athletic horses repetitively participating in two novice jumping classes on consecutive days

**DOI:** 10.3389/fvets.2024.1456733

**Published:** 2024-10-22

**Authors:** Thita Wonghanchao, Onjira Huangsaksri, Kanokpan Sanigavatee, Chanoknun Poochipakorn, Sarisa Chanprame, Sirapatch Wongkosoljit, Wanlapa Chotiyothin, Nontaruj Rattanayanon, Ratsamin Kiawwan, Metha Chanda

**Affiliations:** ^1^Veterinary Clinical Study Program, Faculty of Veterinary Medicine, Kasetsart University, Kamphangsaen, Thailand; ^2^Department of Large Animal and Wildlife Clinical Science, Faculty of Veterinary Medicine, Kasetsart University, Kamphangsaen, Thailand; ^3^Thailand Equestrian Federation, Sports Authority of Thailand, Bangkok, Thailand; ^4^Veterinary Science Program, Faculty of Veterinary Medicine, Kasetsart University, Kamphangsaen, Thailand

**Keywords:** heart rate variability, horse, jumping, repetitive competition, stress response, welfare

## Abstract

**Introduction:**

Animal welfare is of great concern in equestrian sports and has been evaluated in athletic horses competing at different levels. However, the impact of consecutive days of jumping competition and the extent of resultant stress responses remains unclear. To address this point, the present study compared the changes in stress response via heart rate variability (HRV) in horses participating in two national jumping events on consecutive days.

**Methods:**

The study involved six experienced horses equipped with heart rate monitoring devices. HRV variables were measured before, during, and after jumping at 10-min intervals for 60 min on each competition day.

**Results:**

Multiple HRV variables decreased to varying degrees on both days from warm-up until 30 min post-jumping. Meanwhile, the mean heart rate increased during jumping and returned to normal levels at 50 min post-jumping on the first day (for all intervals, *p* < 0.05–0.001), while it remained elevated beyond 60 min post-jumping on the second day (for all intervals, *p* < 0.01–0.001). Additionally, maximum heart rate and respiratory rate were higher on the second day than in the first round during the warm-up phase (*p* < 0.05 for both variables). The proportion of the HRV low-frequency band was higher during riding on the second day (*p* < 0.05), while the proportion of the high-frequency band was reduced during warm-up on the first day (*p* < 0.05) and during course riding on the second (*p* < 0.01). Meanwhile, the sympathetic nervous system index took longer to return to baseline on the second day than on the first.

**Discussion:**

These results suggest that autonomic regulation differed in horses between jumping rounds on two consecutive days, with horses experiencing higher sympathetic activity and potentially increased stress in the second round. This information is important for riders, highlighting the need to be mindful of potential stress that could, at least in part, impact the welfare of horses participating in the same jumping competition on consecutive days.

## 1 Introduction

Equestrian sports such as dressage, eventing, endurance, and jumping are increasingly popular worldwide. International equestrian events are governed by the Fédération Equestre Internationale (FEI) to ensure fairness during the competition and to preserve horse welfare throughout the competition period (1, 2). The FEI Database contains 272,000 horses and nearly 60,000 are registered annually by national federations (3). Athletic horses frequently encounter stressful conditions during training and competition (4). Although horses are well able to perform a variety of exercises of different intensities, diverse factors may compromise performance and welfare, including insufficient or inhumane training (5), mismatching between rider and horse (6) and insufficient rest before repeated competition (7).

Recently, horse welfare in equestrian sports has become a concern to the general public and stakeholders (8). Researchers have proposed various parameters that may reflect welfare in horses, including hormonal release (9, 10), behaviour changes (11, 12), and heart rate variability (HRV) modulation (13, 14). HRV is a short-term fluctuation in the interval between adjacent heartbeats during the cardiac cycle (15, 16). These variations are governed by the autonomic nervous system (ANS), specifically the interplay between the sympathetic nervous system (SNS) and parasympathetic nervous system (PNS) or vagal components (15, 17, 18), known as sympathovagal interaction. An increased HRV reflects the flexibility and adaptability of the body’s biological system to cope with external challenges and maintain its physiological function (15, 19). Modulation in HRV has been used as an indicator of stress responses in horses with cardiac diseases (20, 21), during road transportation (10, 22, 23), and exercise (24–26). HRV has also been used to monitor ANS responses in athletic horses participating in equestrian events such as dressage (9, 27), eventing (28), and jumping (29, 30).

Several HRV variables are applied to indicate sympathetic and vagal activities. Beat-to-beat (RR) intervals and the standard deviation of normal-to-normal intervals (SDNN) reflect the long-term variability of heartbeat, which is influenced by both sympathetic and vagal components (15). In contrast, measurements of the square root of the mean squared differences between successive RR intervals (RMSSD), the number of successive RR interval pairs that differ by more than 50 msec (NN50) and its relative value (pNN50) mirror the short-term variation of heartbeats under the influence of vagal activity (16, 17). A decrease in these time domain variables indicates reduced vagal activity (15, 31, 32). The low-frequency (LF) band modulates under sympathetic and vagal contribution and mirrors the cardiac cycle’s long-term variation. An increased high-frequency (HF) band reflects vagal dominance, contributing to short-term heartbeat variation (15–17). The LF/HF ratio is an indicator of sympathovagal balance (17, 33, 34), with a decreased LF/HF ratio reflecting vagal dominance (16, 35). The standard deviation of the Poincaré plot perpendicular to the line of identity (SD1) is used to determine short-term heart rate variation reflecting vagal activity (17), while the standard deviation of the Poincaré plot along the line of identity (SD2) is an indicator of long-term heart rate variability under sympathetic and vagal influences (16, 17). The SD2/SD1 ratio closely relates to the LF/HF ratio for the determination of ANS balance (16, 36).

Show jumping is growing in popularity, and is organised in international and local competitions worldwide (2). The jumping horse has to clear various obstacles of different heights and difficulties within a limited time to obtain a clear round (2, 37). It has been reported that HRV differs between horses competing in jumping courses with different fence heights, with reduced HRV during the course indicating greater stress (29). However, HRV variables have not been examined in horses participating in jumping courses on two consecutive days. Therefore, this study aimed to compare the HRV modification in horses competing over 2 days at a national jumping event. It was hypothesised that the extent of HRV modulation differs between horses participating in the first and second jumping courses taking place on consecutive days.

## 2 Materials and methods

### 2.1 Horses

Nine healthy warm-blooded horses (three geldings, six mares, aged 10–14 years, weighing 400–500 kg), all trained and experienced in national jumping competitions, were enrolled. The study took place during a national jumping competition at the Thai Polo and Equestrian Club in Pattaya, Thailand. Before the study, all horses underwent physical examination by an equine practitioner to assess vital signs and gait. A normal haemogram was also observed following haematological examination. During the competition, horses were housed in a 4x4x5 m designated stable at the venue. Two to three kilogrammes of commercial pellets were fed three times daily. The horses had free access to hay and water in their stables. The inclusion criteria for horses in this study were: (1) the horse had participated in at least one jumping event during the year before the study; (2) the horse had passed the first inspection according to national jumping regulations; (3) the horse had not received medical or surgical treatment for at least 2 weeks before the study. This study did not cause horses to suffer injury or experience circumstances that compromised their welfare before or during the competition. The horses’ owners signed consent forms before the start of the study. Riders or the person responsible for the horses’ care were able to withdraw the horse at any stage of the study.

### 2.2 Experimental protocol

This study was conducted as a case–control study, requiring the horses to compete in two jumping courses on two consecutive days. Riders were also designated to ride the same horse on both days. The jumping events were run on a 55 × 90 metres silica sand competition arena, containing 10–11 jumping fences with specific heights for each course. The relative humidity and air temperature during the two-day experiment were 85–90% and 32–34°C, respectively. During the experiment, a heart rate monitoring (HRM) device was installed on horses to record RR interval data for HRV determination. The HRM set included an equine belt for riding (Polar Electro Oy, Kempele, Finland), a heart rate sensor (Polar H10) and a sports watch for receiving RR intervals data (Polar Vantage 2). Briefly, horses were equipped with a heart rate sensor attached to the equine belt on the left side of the chest. The sensor was wirelessly connected to the sports watch. The HRM device was installed at least 30 min before the warm-up session and removed 60 min after jumping. Three horses were withdrawn from the study by their riders after obtaining a clear round at the first jumping course. RR interval data from only the six horses who participated in both event days were used for HRV determination in this study.

### 2.3 Data acquisition

RR interval data were recovered from the sports watch using the Polar Flow program.^1^ Kubios premium software (Kubios HRV Scientific)^2^ was then utilised to compute HRV variables from the RR interval data. The premium version of the Kubios program provides automatic artefact correction algorithms, which are validated and produce more accurate time series evaluations than the standard version (38). The additional automatic noise detection function removes unpredictable noise in long-term RR interval recording where the signal may not be high quality throughout.^3^ Trend components were modified using smoothness priors at 500 ms. The autonomic noise correction was set at a medium level. The following HRV variables were computed:

*Time domain analysis*: heart rate (**HR**), beat-to-beat (**RR**) intervals, standard deviation of the normal-to-normal intervals (**SDNN**), square root of the mean squared differences between successive RR intervals (**RMSSD**), relative number of successive RR interval pairs that differ by more than 50 msec (**pNN50**), triangular interpolation of normal-to-normal intervals (**TINN**) and RR triangular index.

*Frequency domain analysis*: very-low-frequency (**VLF**) (by default 0–0.04 Hz), low-frequency (**LF**) (by default 0.04–0.15 Hz), high-frequency (**HF**) (by default 0.15–0.4 Hz), **LF/HF ratio** and respiratory rate (**RESP**).

*Nonlinear analysis*: standard deviation of the Poincaré plot perpendicular to the line of identity (**SD1**), standard deviation of the Poincaré plot along the line of identity (**SD2**) and SD2/SD1 ratio.

*Autonomic nervous system (ANS) index*: stress index, parasympathetic nervous system (**PNS**) index and sympathetic nervous system (**SNS**) index.

The HRV variables were reported as 15 min at rest (control), warm-up period, course jumping period, and 10-min periods at 10, 20, 30, 40, 50, and 60 min post-jumping.

### 2.4 Data analysis

Data were analysed using GraphPad Prism version 10.2.3 (GraphPad Software Inc., San Diego, USA). Due to missing data during the measurement, a mixed-effects model (restricted maximum likelihood; REML) was applied to evaluate the effects of group, time, and group-by-time on changes in HRV variables in horses on both jumping days. Dunnett’s multiple comparisons test was used to estimate the differences in HRV variables within the group compared to those before the competition (control) and between groups at given time points. Due to the normal distribution of the data confirmed by the Shapiro–Wilk test, a paired *t*-test was used to calculate the differences in competition speed, warm-up and course jumping periods between groups. Data were expressed as mean ± SEM. The statistical significance was set at *p* < 0.05.

## 3 Results

### 3.1 Warm-up period, riding speed, and riding duration during jumping on both days

The parameters and competition results from the first and second jumping days are shown in Table 1. The horses participated in jumping courses with a fence height of 70–120 cm in the first and 70–130 cm in the second courses, respectively. Horses 1 and 6 jumped an increased fence height in the second course, while the other horses (horses 2–5) jumped a similar height in both jumping courses. On both days, a similar warm-up period (63.13 ± 8.10 vs. 56.96 ± 8.70 min, *p* = 0.1827), riding speed (3.72 ± 0.24 vs. 3.53 ± 0.29 m/s, *p* = 0.6098) and riding duration (71.63 ± 6.95 vs. 74.91 ± 7.68 s, *p* = 0.3307) were observed. Only horse 1 achieved two clear rounds, while horses 2 and 4 were eliminated due to refusal in both courses. Likewise, the performance regarding total penalties of horses 3, 5 and 6 was similar on both days.

**Table 1:** Parameters and competition results of horses participating in 2 days of a jumping competition.

Horses	Fence height (cm)	Average speed (m/s)	Jump faults (points)	Time taken (sec)	Time faults (points)	Total penalty
First jumping day						
No. 1	70	4.02	0	52.83	0	0
No. 2	100	3.45	–	–	–	E
No. 3	70	3.47	8	84.25	18	26
No. 4	100	4.76	–	–	–	E
No. 5	70	3.08	4	69.84	0	4
No. 6	120	3.56	16	79.60	0	16
Second jumping day						
No. 1	90	3.16	0	58.92	0	0
No. 2	100	3.94	–	–	–	E
No. 3	70	4.65	4	93.91	24	28
No. 4	100	3.71	–	–	–	E
No. 5	70	2.86	0	66.84	0	0
No. 6	130	2.86	16	79.98	0	16

### 3.2 Heart rate variability

#### 3.2.1 Time domain analysis

Time, group and group-by-time affected the modulation of peak HR (*p* < 0.0001, *p* = 0.0364 and *p* = 0.0049, respectively), while group-by-time and time affected changes in minimum HR (*p* < 0.0001 and *p* = 0.0084), mean HR (*p* < 0.0001 for both effects) and mean RR intervals (*p* < 0.0001 and *p* = 0.0178). SDNN, RMSSD, pNN50, TINN, and RR triangular index were associated with time (*p* < 0.0001 for all variables; Tables 2, 3).

**Table 2:** Time-domain analysis of HRV in horses participating in 2 days of a national jumping competition.

Periods	Classes	Time domain results			
		Mean HR (beats/min)	Min HR (beats/min)	Max HR (beats/min)	Mean RR (ms)
Control	1^st^	35.50 ± 2.38	31.00 ± 1.95	51.43 ± 4.21	1818.17 ± 107.10
	2^nd^	33.17 ± 1.58	29.17 ± 1.25	52.17 ± 5.38	1837.83 ± 91.12
Warm-up	1^st^	66.83 ± 7.44^a^	37.67 ± 5.40	125.67 ± 11.31^b,x^	963.50 ± 121.47^b^
	2^nd^	87.50 ± 7.34^b^	34.17 ± 2.27^a^	166.17 ± 8.58^d,y^	716.17 ± 73.49^c^
Course riding	1^st^	114.00 ± 22.00	100.00 ± 10.00	124.00 ± 30.00	545.00 ± 105.00
	2^nd^	162.50 ± 10.71^c^	132.67 ± 10.76^c^	178.50 ± 11.51^c^	378.17 ± 26.25^d^
10 min post-jumping	1^st^	74.60 ± 6.06^b^	64.40 ± 6.27^b^	91.00 ± 6.80^c^	826.20 ± 69.81^b^
	2^nd^	76.00 ± 4.32^c^	60.67 ± 3.14^c^	120.67 ± 17.08	802.83 ± 45.21^c^
20 min post-jumping	1^st^	55.40 ± 3.64^c^	45.40 ± 1.94^b^	82.00 ± 9.06	1099.80 ± 66.81^b^
	2^nd^	57.33 ± 2.74^d^	44.50 ± 2.78^b^	77.67 ± 2.32^a^	1056.83 ± 46.59^c^
30 min post-jumping	1^st^	48.25 ± 3.90^b^	40.50 ± 2.06^a^	68.75 ± 8.22	1271.00 ± 107.54^b^
	2^nd^	49.33 ± 3.48^b^	41.83 ± 2.81^b^	65.83 ± 4.35	1246.50 ± 83.29^c^
40 min post-jumping	1^st^	45.50 ± 3.07^a^	37.50 ± 2.25^a^	66.50 ± 5.95	1337.00 ± 88.59
	2^nd^	47.60 ± 4.26^a^	41.20 ± 2.54^b^	71.00 ± 3.85	1297.20 ± 105.33^b^
50 min post-jumping	1^st^	43.80 ± 1.91^a^	36.80 ± 1.69^a^	58.40 ± 5.60	1384.20 ± 65.07
	2^nd^	46.00 ± 3.56^a^	40.20 ± 2.69^a^	62.80 ± 6.30	1330.80 ± 95.15^b^
60 min post-jumping	1^st^	42.40 ± 2.48	36.60 ± 1.47^a^	62.80 ± 8.67	1443.20 ± 91.47
	2^nd^	48.20 ± 2.33^b^	40.00 ± 2.37^a^	70.80 ± 7.97	1264.20 ± 59.53^b^
Effects (*p*-value)	Time	<0.0001*	<0.0001*	<0.0001*	<0.0001*
	Group	0.1075	0.3645	0.0364*	0.4770
	Interaction	<0.0001*	0.0084*	0.0049*	0.0178*

*Indicate significant effects of time, group, and group-by-time (interaction). a, b, c and d indicate statistical significance at *p* < 0.05, 0.01, 0.001, and 0.0001 within the group compared to the control value. x and y indicate statistical differences of a pair of comparisons between groups at given time points. HRV, heart rate variability; RR, beat-to-beat interval; HR, heart rate; SDNN, standard deviation of normal-to-normal RR intervals; RMSSD, root mean square of successive RR interval differences. Recovery post-jumping.

**Table 3:** Time-domain analysis of HRV in horses participating in 2 days of a national jumping competition.

Periods	Classes	Time domain results				
		SDNN (ms)	RMSSD (ms)	pNN50 (%)	TINN (ms)	RR triangular index
Control	1^st^	66.40 ± 11.41	67.07 ± 13.09	30.99.5.89	379.83 ± 64.68	13.26 ± 1.91
	2^nd^	61.18 ± 6.80	54.12 ± 4.00	25.87 ± 3.60	339.33 ± 34.15	11.26 ± 1.15
Warm-up	1^st^	40.55 ± 5.22^c^	25.15 ± 3.98^b^	6.96 ± 2.47^c^	301.33 ± 30.83	7.91 ± 1.44^a^
	2^nd^	31.95 ± 4.79^c^	17.73 ± 4.00^b^	3.28 ± 1.65^c^	284.83 ± 34.25	5.89 ± 0.76^a^
Course riding	1^st^	6.85 ± 1.25^d^	4.40 ± 1.60^b^	0.00 ± 0.00^b^	30.00 ± 15.00^c^	2.09 ± 0.59^b^
	2^nd^	10.07 ± 1.50^d^	3.92 ± 0.15^b^	0.00 ± 0.00^b^	43.33 ± 5.77^c^	3.25 ± 0.37^b^
10 min post-jumping	1^st^	26.98 ± 9.98^a^	19.20 ± 8.60^b^	2.24 ± 1.46^c^	131.20 ± 39.34^b^	4.63 ± 0.88^b^
	2^nd^	22.30 ± 6.21^a^	16.30 ± 5.06^b^	3.53 ± 2.48^c^	147.33 ± 39.18^b^	4.46 ± 0.97^b^
20 min post-jumping	1^st^	40.48 ± 4.47^a^	33.72 ± 4.28^a^	13.50 ± 4.13^b^	193.20 ± 27.15^a^	8.41 ± 1.22
	2^nd^	44.77 ± 7.68^a^	34.58 ± 5.18^a^	13.34 ± 3.54^b^	247.17 ± 41.33^a^	10.60 ± 1.95
30 min post-jumping	1^st^	56.35 ± 7.76	46.03 ± 5.43	22.40 ± 4.93	311.00 ± 39.74	12.67 ± 1.85
	2^nd^	48.60 ± 6.48	40.85 ± 5.95	16.75 ± 4.29	268.67 ± 39.52	10.20 ± 1.27
40 min post-jumping	1^st^	73.05 ± 12.44	62.45 ± 14.01	30.54 ± 6.95	346.50 ± 58.38	14.15 ± 1.74
	2^nd^	57.38 ± 11.49	47.30 ± 10.70	19.32 ± 6.07	313.20 ± 50.17	10.52 ± 2.16
50 min post-jumping	1^st^	71.82 ± 7.11	61.88 ± 6.54	35.29 ± 4.15	326.80 ± 32.72	13.91 ± 1.46
	2^nd^	61.72 ± 14.40	53.36 ± 11.85	24.69 ± 7.89	312.60 ± 71.56	13.03 ± 2.75
60 min post-jumping	1^st^	76.36 ± 7.24	77.06 ± 14.61	39.19 ± 7.04	358.40 ± 41.74	13.71 ± 2.65
	2^nd^	83.48 ± 15.79	67.00 ± 13.25	32.66 ± 7.41	394.60 ± 65.52	17.53 ± 3.07
Effects (*p*-value)	Time	<0.0001*	<0.0001*	<0.0001*	<0.0001*	<0.0001*
	Group	0.7569	0.4132	0.3571	0.8456	0.9401
	Interaction	0.6004	0.9258	0.5899	0.5598	0.1544

*Indicate significant effects of time, group, and group-by-time (interaction). a, b and c indicate statistical significance at *p* < 0.05, 0.01 and 0.001 within the group compared to the control value. HRV, heart rate variability; NN50, number of successive RR interval pairs that differ by more than 50 msec; pNN50, relative number of successive RR interval pairs that differ by more than 50 msec; TINN, triangular interpolation of normal-to-normal intervals; RR, beat-to-beat interval.

A significant increase in mean HR was detected during warm-up in horses in the first jumping course. During the jumping period, the increase in mean HR was not significant (*p* = 0.2379), but an increased mean HR was again detected at 10–50 min post-jumping (for all intervals, *p* < 0.05–0.001) in horses on the first day. On the second jumping day, mean HR increased during the warm-up period and the increase over baseline lasted until 60 min post-jumping (for all intervals, *p* < 0.05–0.001). Mean HR was higher in horses in the second than the first jumping course, but this difference was insignificant (*p* = 0.0762). Minimum HR rose at 10–60 min post-jumping in horses on the first day (*p* < 0.05–0.01), but remained increased from the warm-up period until 60 min post-jumping on the second (*p* < 0.05–0.001). Peak HR increased during the warm-up period (first course: *p* < 0.01, second course: *p* < 0.0001), and values were higher during the second than the first course (*p* < 0.05). Moreover, compared to the control period, an increased peak HR during jumping was detected only during the second course (*p* < 0.001). A significant decrease in mean RR interval was measured during the warm-up period before the first jumping course. Although there was an insignificant decrease in the mean RR interval during jumping (*p* = 0.1041), a considerable reduction in the mean RR interval was subsequently registered at 10–30 min post-jumping following the first course (*p* < 0.01 for all mentioned periods). On the second day, the mean RR interval decreased during the warm-up period and remained decreased until 60 min post-jumping (for all intervals, *p* < 0.01–0.0001). On both jumping days, SDNN and RMSSD decreased during warm-up (*p* < 0.001 and *p* < 0.01, respectively) and then plunged to the lowest value during jumping (*p* < 0.0001 and *p* < 0.01, respectively). They eventually rose to reach values similar to baseline at 30 min post-jumping (*p* < 0.05–0.01 for both variables; Table 2). Although only pNN50 began to decrease in the warm-up period, pNN50 and TINN decreased during jumping and remained lower until 20 min post-jumping (pNN50, *p* < 0.01–0.001; TINN, *p* < 0.05–0.001). The RR triangular index declined during warm-up (*p* < 0.05–0.01), jumping (*p* < 0.01) and 10 min post-jumping (*p* < 0.01; Table 3).

### 3.3 Frequency domain analysis

There was a group-by-time and time effect on the modification of the proportion of LF (*p* = 0.0016 and *p* = 0.0495, respectively), while the change in HF proportion was only affected by group-by-time (*p* = 0.0169). There was an independent group effect for RESP (*p* = 0.0229). Modulation in VLF % was associated with time (*p* = 0.0132, respectively); meanwhile, the LF/HF ratio was unaffected by the effects of group-by-time, group or time (Table 4).

**Table 4:** Frequency domain analysis of HRV in horses participating in 2 days of a national jumping competition.

Periods	Classes		Frequency domain results			
		VLF (%)	LF (%)	HF (%)	LF/HF ratio	RESP (Hz)
Control	1^st^	0.25 ± 0.03	0.56 ± 0.03	0.19 ± 0.02	3.13 ± 0.48	0.195 ± 0.015
	2^nd^	0.34 ± 0.03	0.53 ± 0.03	0.12 ± 0.02	4.94 ± 0.77	0.188 ± 0.014
Warm-up	1^st^	0.32 ± 0.05	0.60 ± 0.05	0.08 ± 0.0^a^	7.88 ± 1.55^a^	0.165 ± 0.027^x^
	2^nd^	0.28 ± 0.02	0.65 ± 0.02	0.07 ± 0.02	10.62 ± 1.91^a^	0.248 ± 0.012^y^
Course riding	1^st^	0.33 ± 0.29	0.32 ± 0.02^x^	0.37 ± 0.34	4.36 ± 3.96	0.245 ± 0.165
	2^nd^	0.40 ± 0.07	0.56 ± 0.08^y^	0.04 ± 0.0^b^	33.22 ± 14.23	0.312 ± 0.040^a^
10 min post-jumping	1^st^	0.28 ± 0.04	0.56 ± 0.06	0.16 ± 0.04	6.58 ± 3.91	0.180 ± 0.047
	2^nd^	0.27 ± 0.06	0.57 ± 0.05	0.16 ± 0.03	4.19 ± 0.93	0.302 ± 0.014^b^
20 min post-jumping	1^st^	0.12 ± 0.05^b^	0.66 ± 0.05	0.22 ± 0.06	3.92 ± 0.75	0.184 ± 0.028
	2^nd^	0.14 ± 0.03^b^	0.67 ± 0.05	0.20 ± 0.04	4.30 ± 1.01	0.235 ± 0.020
30 min post-jumping	1^st^	0.14 ± 0.02^b^	0.69 ± 0.03	0.17 ± 0.03	4.48 ± 0.94	0.225 ± 0.010
	2^nd^	0.15 ± 0.03^b^	0.64 ± 0.06	0.21 ± 0.06	4.81 ± 1.46	0.223 ± 0.010
40 min post-jumping	1^st^	0.18 ± 0.05^a^	0.67 ± 0.04	0.16 ± 0.04	5.30 ± 1.62	0.200 ± 0.007^x^
	2^nd^	0.24 ± 0.06^a^	0.57 ± 0.04	0.19 ± 0.05	3.95 ± 0.87	0.232 ± 0.006^y^
50 min post-jumping	1^st^	0.18 ± 0.02^a^	0.68 ± 0.02	0.14 ± 0.03	6.78 ± 2.43	0.178 ± 0.036
	2^nd^	0.14 ± 0.02^a^	0.66 ± 0.05	0.19 ± 0.05	4.28 ± 0.98	0.230 ± 0.008
60 min post-jumping	1^st^	0.18 ± 0.07	0.62 ± 0.05	0.20 ± 0.05	4.02 ± 1.10	0.196 ± 0.033
	2^nd^	0.18 ± 0.07	0.68 ± 0.06	0.13 ± 0.02	5.60 ± 1.09	0.234 ± 0.007
Effects (*p*-value)	Time	0.0132*	0.0016*	0.2201	0.2196	0.2848
	Group	0.5371	0.6248	0.1448	0.2108	0.0229*
	Interaction	0.8679	0.0495*	0.0358*	0.1085	0.1721

*Indicate significant effects of time, group, and group-by-time (interaction). a and b indicate statistical significance at *p* < 0.05, 0.01 within the group compared to the control value. x and y indicate statistical differences of a pair of comparisons between groups at given time points. HRV, heart rate variability; VLF, very-low-frequency band, by default 0–0.04 Hz; LF: low-frequency band, by default 0.04–0.15 Hz; HF, high-frequency band, by default 0.15–0.4 Hz; RESP, respiration rate.

The VLF proportion decreased at 20–50 min post-jumping (for all intervals, *p* < 0.05–0.01). Even though the LF percentage did not change throughout the study over both jumping days, it was higher in horses during jumping in the second than in the first course (*p* < 0.05). The HF percentage decreased during the warm-up period in horses before the first course (*p* < 0.05) and during jumping in the second course (*p* < 0.01), while the LF/HF ratio rose only during warm-up (*p* < 0.05). There was no change in RESP throughout the first day, while the RESP increased during jumping (*p* < 0.05) and 10 min post-jumping (*p* < 0.01) on the second day. Moreover, RESP during warm-up and 40 min post-jumping was higher on the second than the first day (*p* < 0.05 for both periods; Table 4).

### 3.4 Nonlinear analysis and ANS index

The SD1, SD2, SD2/SD1 ratio, stress index and PNS index changes were associated with time (*p* < 0.0001 for all variables). In contrast, the SNS index change was associated with group-by-time (*p* = 0.0313) and time (*p* < 0.0001; Table 5).

**Table 5:** Nonlinear analysis and autonomic nervous system (ANS) index of HRV in horses participating in 2 days of a national jumping competition.

Periods	Classes	Nonlinear results			ANS index		
		SD1 (ms)	SD2 (ms)	SD2/SD1 ratio	Stress index	PNS index	SNS index
Control	1^st^	47.50 ± 9.26	80.52 ± 13.84	1.72 ± 0.16	4.97 ± 0.78	4.29 ± 0.68	−2.64 ± 0.22
	2^nd^	38.30 ± 3.64	77.08 ± 9.83	2.04 ± 0.22	4.90 ± 0.37	4.40 ± 0.51	−2.76 ± 0.15
Warm-up	1^st^	17.80 ± 2.82^b^	54.28 ± 6.84	3.19 ± 0.25^c^	9.20 ± 1.43^b^	−0.38 ± 0.65^d^	0.17 ± 0.70^a^
	2^nd^	12.57 ± 2.84^b^	43.32 ± 6.22	3.74 ± 0.29^c^	11.22 ± 1.50^b^	−1.75 ± 0.46^d^	1.85 ± 0.68^b^
Course riding	1^st^	3.20 ± 1.20^b^	8.65 ± 2.65^c^	3.47 ± 2.11^a^	54.55 ± 10.85^d^	−2.98 ± 0.87^d^	11.03 ± 0.60^a^
	2^nd^	2.77 ± 0.10^b^	13.90 ± 2.17^c^	5.06 ± 0.84^a^	53.15 ± 5.87^d^	−4.25 ± 0.19^d^	15.82 ± 1.99^b^
10 min post-jumping	1^st^	13.68 ± 6.17^b^	35.68 ± 12.99^a^	3.00 ± 0.55	19.96 ± 4.08^b^	−1.28 ± 0.55^d^	2.30 ± 0.94^a^
	2^nd^	11.50 ± 3.59^b^	29.30 ± 8.06^a^	2.65 ± 0.28	17.90 ± 3.84^b^	−1.29 ± 0.34^d^	2.05 ± 0.76^b^
20 min post-jumping	1^st^	23.88 ± 3.03^a^	51.92 ± 5.70^a^	2.21 ± 0.12	9.82 ± 1.04^b^	0.54 ± 0.31^d^	−0.55 ± 0.32^b^
	2^nd^	24.50 ± 3.67^a^	58.37 ± 10.30^a^	2.31 ± 0.15	8.98 ± 1.66^b^	0.37 ± 0.30^d^	−0.55 ± 0.39^b^
30 min post-jumping	1^st^	32.58 ± 3.84	72.65 ± 10.35	2.21 ± 0.11	6.25 ± 0.58^a^	1.63 ± 0.43^d^	−1.56 ± 0.21^b^
	2^nd^	28.92 ± 4.22	62.00 ± 8.63	2.17 ± 0.24	7.80 ± 1.33^a^	1.40 ± 0.45^d^	−1.27 ± 0.35^a^
40 min post-jumping	1^st^	44.20 ± 9.94	93.25 ± 15.03	2.19 ± 0.18	5.50 ± 0.71	2.37 ± 0.44^b^	−1.86 ± 0.17
	2^nd^	33.48 ± 7.58	73.50 ± 14.85	2.22 ± 0.28	7.26 ± 1.64	1.79 ± 0.61^b^	−1.45 ± 0.42^a^
50 min post-jumping	1^st^	43.86 ± 4.64	91.58 ± 9.62	2.12 ± 0.21	5.52 ± 0.54	2.57 ± 0.36^b^	−1.98 ± 0.17
	2^nd^	37.78 ± 8.39	78.38 ± 18.83	1.99 ± 0.24	7.52 ± 2.30	2.13 ± 0.55^b^	−1.54 ± 0.42^a^
60 min post-jumping	1^st^	55.14 ± 10.86	88.84 ± 11.94	1.85 ± 0.34	4.98 ± 0.52	3.31 ± 0.83	−2.23 ± 0.24
	2^nd^	47.46 ± 9.39	107.96 ± 20.56	2.27 ± 0.19	5.40 ± 1.01	2.16 ± 0.42	−1.71 ± 0.21^c^
Effects (*p*-value)	Time	<0.0001*	<0.0001*	0.0058*	<0.0001*	<0.0001*	<0.0001*
	Group	0.4024	0.8962	0.2589	0.9951	0.3499	0.2305
	Interaction	0.9294	0.4158	0.5511	0.9685	0.3158	0.0313*

*Indicate significant effects of time, group, and group-by-time (interaction). a, b, c and d indicate statistical significance at *p* < 0.05, 0.01, 0.001, and 0.0001 within the group compared to the control value. SD1, standard deviation of the Poincaré plot perpendicular to the line of identity; SD2, standard deviation of the Poincaré plot along the line of identity; PNS, parasympathetic nervous system; SNS, sympathetic nervous system.

There was a decrease in SD1 during the warm-up period (*p* < 0.01), and a decrease in both SD1 and SD2 was observed during jumping and up to 20 min post-jumping (SD1, *p* < 0.05–0.01; SD2, *p* < 0.05–0.001). The SD2/SD1 ratio increased during warm-up (*p* < 0.001) and jumping (*p* < 0.05). The stress index increased during warm-up (*p* < 0.01) and reached the highest value during jumping (*p* < 0.0001). Despite a gradual decrease, the stress index remained higher than the control until 30 min post-jumping. The PNS index decreased during warm-up, substantially declined to the lowest value during jumping, and remained below the baseline during 50 min post-jumping (for all intervals, *p* < 0.01–0.0001). The SNS index increased during warm-up and remained higher than the control up to 30 min post-jumping in horses on the first day (*p* < 0.05–0.01), and up to 60 min post-jumping in horses on the second (*p* < 0.05–0.001). The SNS index during jumping was numerically higher in horses during the second course compared to the first, but this difference was not significant (*p* = 0.0628; Table 5).

## 4 Discussion

In this study, the modulation of HRV variables in horses competing in national jumping events on consecutive days was compared. The study yielded several significant findings: (1) there was an interaction between event day and time for changes in mean HR, minimum HR, peak HR, and HRV variables such as mean RR intervals, LF percentage, HF percentage, and SNS index; (2) compared to baseline values, the mean, minimum, and peak HR values during jumping remained unchanged on the first day but increased on the second; (3) HRV decreased during the warm-up period and reached the lowest values during jumping; (4) mean RR intervals and the SNS index returned to baseline earlier in horses on the first day compared to the second; (5) RESP increased during jumping and 10 min post-jumping only on the second day; and (6) the higher peak HR and RESP during warm-up and the LF percentage during jumping were only detected in horses on the second day. These results indicate that horses exhibited a higher sympathetic tone while participating on the second jumping day compared to the first.

It is interesting to note that the competition results for the horse-rider combinations were quite consistent across both rounds. It is possible that the overall performance of the individual horses played a significant role in the observed results (39, 40). Research has shown that horses engaged in different activities exhibit distinct behaviours and emotional responses (41). For instance, it is believed that horses bred for jumping are genetically less sensitive to frightening stimuli compared to dressage horses (42). By familiarising horses with potentially scary situations through training, they may display minimal or absent responses to such stimuli (43). Unexpectedly, horses 2 and 4 were eliminated in both jumping courses. It is possible that these horses exhibited negative fear-related responses to potential challenges during competition, similar to previously reported findings (43).

Ensuring a thorough warm-up session before exercise is crucial to prepare the horse for equestrian competitions (44). Its primary goals include improving muscle function, minimising the risk of injury, and maximising performance (45). Research has found that in dressage competitions, warm-up periods tend to be longer for more complex events (46). However, when it comes to jumping competitions, the length of the warm-up period remains consistent across different days of the competition (47, 48). Consistent with this finding, our study also observed no difference in the warm-up period between jumping courses on consecutive competition days. Nonetheless, the present results contrast with a study by Tranquille et al., which did not detect differences in average and peak heart rate during warm-up periods between days (48). The current findings revealed a trend towards higher average heart rate and a significantly higher peak HR in horses during the warm-up period for the second course compared to the first. This increase in heart rate and respiration may partly be attributed to the rider exerting more effort to prepare the horse for the second course following an unsuccessful ride on the first day.

Interestingly, during the second jumping day, there was a notable increase in the average, minimum, and peak HR from the warm-up to the course itself, compared to the first day where the increases were insignificant. The heightened HR could indicate excitement (49, 50), increased physical activity (29, 51), or stress (52, 53). This rise in HR during jumping may have implications for multi-day competitions (54). Conversely, when a well-matched horse and rider pair is involved, the horse’s HR decreases during the ride (6). Since matching pairs participated on both days, the significant increase in all HR parameters during jumping on the second day likely signifies increased effort by the horse. The delayed decrease in mean HR beyond 60 min post-jumping after the second course, compared to a return to baseline at 50 min post-jumping after the first, suggests a higher exercise intensity on the second day.

The literature widely acknowledges that HRV decreases in horses during exercise (24, 25), particularly in the context of equestrian competitions (9, 29). This decrease is indicative of reduced vagal activity and increased sympathetic tone (15, 17). The present study’s findings aligned with this understanding, as decreased HRV parameters such as mean RR, SDNN, RMSSD, pNN50, TINN, RR triangular index, SD1, and SD2 were observed during riding periods and up to 20 min post-jumping, signalling sympathetic dominance in horses during these activities. Time of the day has been reported as a contributing factor, as circadian variation means parasympathetic activity is relatively increased at night (55). Accordingly, this factor should be controlled for to ensure accurate HRV analysis (15). The jumping courses in the present study were officially scheduled for approximately 8.00–11.30 AM to avoid hot and humid weather in the afternoon. Hence, the recorded activity occurred at almost the same time across the two-day experiment and an effect of circadian rhythm can be disregarded. Specific changes in mean RR intervals and contributions of the LF and HF bands were also noted between jumping courses, demonstrating distinct autonomic responses in horses. It has been reported that sympathetic and parasympathetic components contribute to the LF band, while the vagal component, in conjunction with respiration rate, affects the change in the HF band (15–17). The higher sympathetic dominance observed during the second jumping course, as evidenced by the increased LF and decreased HF bands, implies that horses experienced greater stress during the second course than during the first. Furthermore, the sustained increase in the SNS index beyond 60 min post-jumping provides additional evidence for heightened sympathetic activity in horses on the second jumping day. This suggests that horses may experience more stress when participating in two jumping courses on consecutive days, particularly during the second event.

While this study offers valuable insights into the autonomic responses in horses participating in consecutive two-day jumping competitions, there are still important unanswered questions. For example, it is unclear to what extent the autonomic response may vary in horses participating for more than 2 days during the same jumping event. Additionally, further investigation is needed to understand how HRV is modulated in horses repeatedly participating in international events with higher fence heights. Another area that requires more research is the interaction between horses and riders, and how this may lead to distinct modifications of HRV variables when repeatedly competing in jumping courses. This study was limited by the small number of horses jumping at the same fence height. Additionally, the variation in fence heights among the courses caused a significant deviation in the HRV values within the group. It is important to note that the high humidity during the experiment may have influenced the HRV modulation, potentially in conjunction with stress or anxiety in the horses. Therefore, any modifications in HRV observed in this study should be interpreted with caution. Lastly, due to concerns from the riders, we were unable to perform blood sampling during the event, preventing the measurement of haematological parameters and stress hormone modulation for this study. These are important considerations for future research in this area.

## 5 Conclusion

The autonomic responses of horses participating in jumping competitions show significant differences between two consecutive days. Horses participating in the second day repeatedly exhibit increased sympathetic activity, indicating higher stress levels than in the first round. These findings shed light on the autonomic responses of horses competing on consecutive days, emphasising the importance of recognising potential stress that could impact the welfare of horses participating in such events.

## Data Availability

The raw data supporting the conclusions of this article will be made available by the authors, without undue reservation.

## Footnotes

^1^ https://flow.polar.com/

^2^ https://www.kubios.com/hrv-premium/

^3^ https://www.kubios.com/downloads/Kubios_HRV_Users_Guide.pdf
